# Polymorphism of 2-(5-benzyl-6-oxo-3-phenyl-1,6-di­hydro­pyridazin-1-yl)acetic acid with two monoclinic modifications: crystal structures and Hirshfeld surface analyses

**DOI:** 10.1107/S2056989020002406

**Published:** 2020-02-25

**Authors:** Said Daoui, Cemile Baydere, Tarik Chelfi, Fouad El Kalai, Necmi Dege, Khalid Karrouchi, Noureddine Benchat

**Affiliations:** a Laboratory of Applied Chemistry and Environment (LCAE), Faculty of Sciences, Mohamed I University, 60000 Oujda, Morocco; b Department of Physics, Faculty of Arts and Sciences, Ondokuz Mayıs University, 55139-Samsun, Turkey; c Laboratory of Medicinal Chemistry, Faculty of Medicine and Pharmacy, University, Mohammed V, Rabat, Morocco

**Keywords:** crystal structure, polymorphism, pyridazine, Hirshfeld surface analysis

## Abstract

Two polymorphs of the title compound, C_19_H_16_N_2_O_3_, were obtained from ethano­lic (polymorph **I**) and methano­lic solutions (polymorph **II**), respectively. Both polymorphs crystallize in the monoclinic system with four formula units per cell and a complete mol­ecule in the asymmetric unit. The main difference between the mol­ecules of (**I**) and (**II**) is the reversed position of the hy­droxy group of the carb­oxy­lic function. All other conformational features are found to be similar in the two mol­ecules. The different orientation of the OH group results in different hydrogen-bonding schemes in the crystal structures of (**I**) and (**II**). Whereas in (**I**) inter­molecular O—H⋯O hydrogen bonds with the pyridazinone carbonyl O atom as acceptor generate chains with a *C*(7) motif extending parallel to the *b-*axis direction, in the crystal of (**II**) pairs of inversion-related O—H⋯O hydrogen bonds with an *R*_2_^2^(8) ring motif between two carb­oxy­lic functions are found. The inter­molecular inter­actions in both crystal structures were analysed using Hirshfeld surface analysis and two-dimensional fingerprint plots.

## Abstract 2

The mol­ecules of the two title polymorphs mainly differ in the orientation of the carb­oxy­lic OH group that results in different packing features.

## Chemical context   

Pyridazin-3(2*H*)-ones are an important family of heterocycles because of their great chemical reactivity (Chelfi *et al.*, 2015 ▸; Zarrouk *et al.*, 2010 ▸), with new products reported recently (Chakraborty *et al.*, 2018 ▸; El Kalai *et al.*, 2019*a* ▸). In addition, the importance of pyridazinones in medicinal chemistry has increased in recent years thanks to their pharmacological properties, including anti­cancer (Yarden & Caldes, 2013 ▸), anti-hypertensive (Siddiqui *et al.*, 2011 ▸), anti­bacterial (Akhtar *et al.*, 2016 ▸), anti-HIV (Livermore *et al.*, 1993 ▸), anti-inflammatory (Singh *et al.*, 2017 ▸), anti­depressant (Boukharsa *et al.*, 2016 ▸), anti-convulsant (Partap *et al.*, 2018 ▸) and cardiotonic (Costas *et al.*, 2015 ▸) activities. Several pyridazinone-based products are already present in the pharmaceutical market such as Minaprine (Sotelo *et al.*, 2003 ▸), Aza­nrinone (Mahmoodi *et al.*, 2014 ▸), Indolidan (Abouzid *et al.*, 2008 ▸) and Levosimendan (Archan & Toller, 2008 ▸).

In a continuation of our recent work on the synthesis and crystal structures of new pyridazin-3(2*H*)-one derivatives (El Kalai *et al.*, 2019*b* ▸; Daoui *et al.*, 2019*a* ▸,*b* ▸), we report here the synthesis, crystal structure and polymorphism of 2-(5-benzyl-6-oxo-3-phenyl­pyridazin-1(*6H*)-yl)acetic acid, which is going to be subjected to further pharmacological investigations.
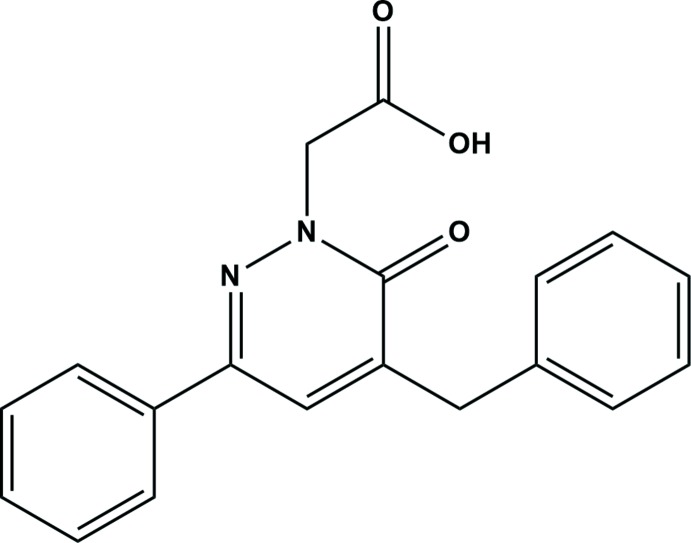


## Structural commentary   

**Figure 1:**
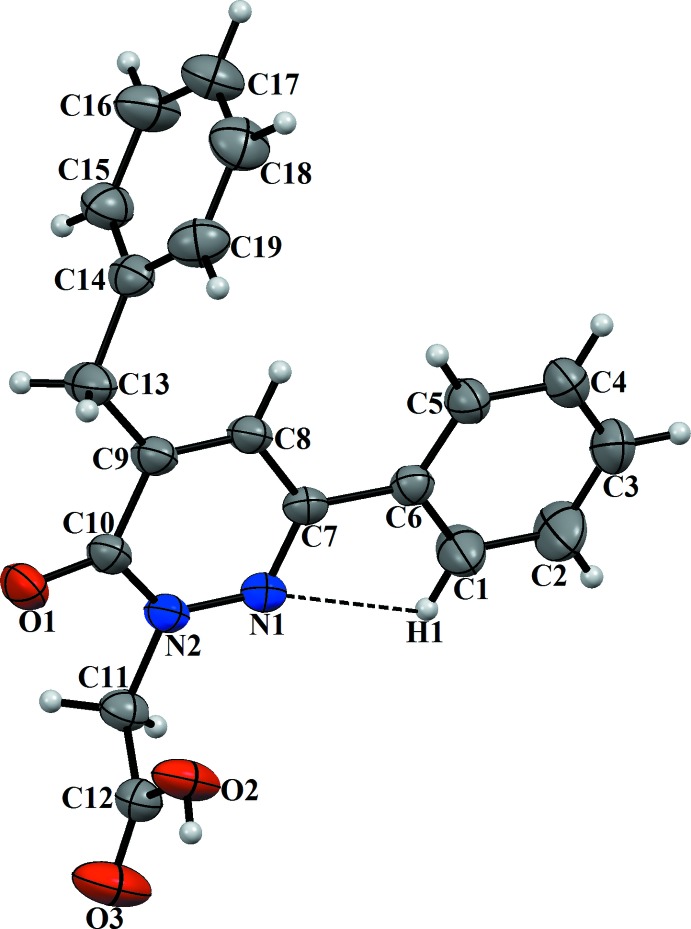
The mol­ecular structure of (**I**) with displacement ellipsoids drawn at the 30% probability level.

**Figure 2:**
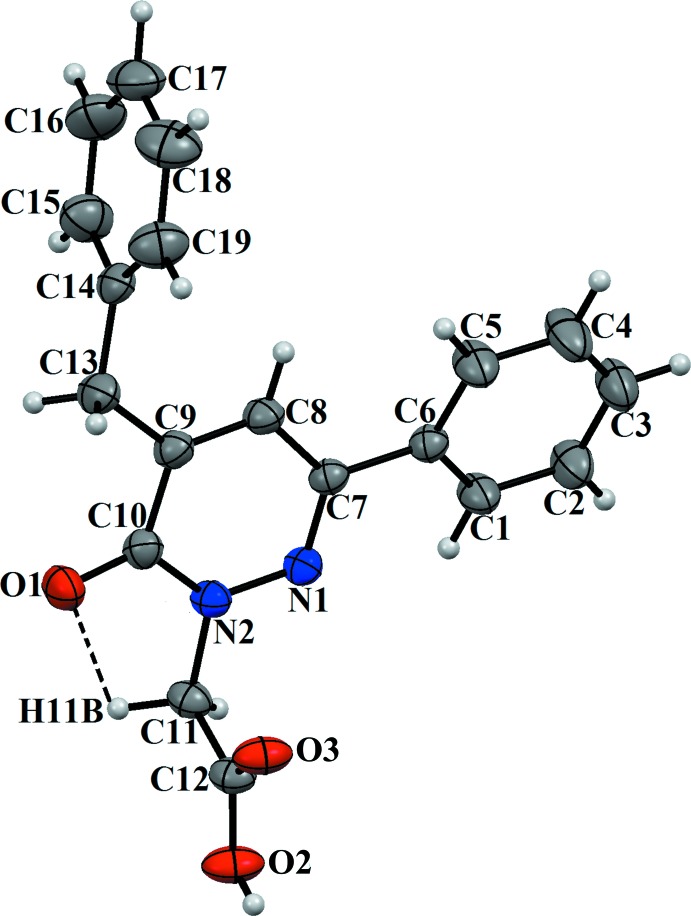
The mol­ecular structure of (**II**) with displacement ellipsoids drawn at the 30% probability level.

**Figure 3:**
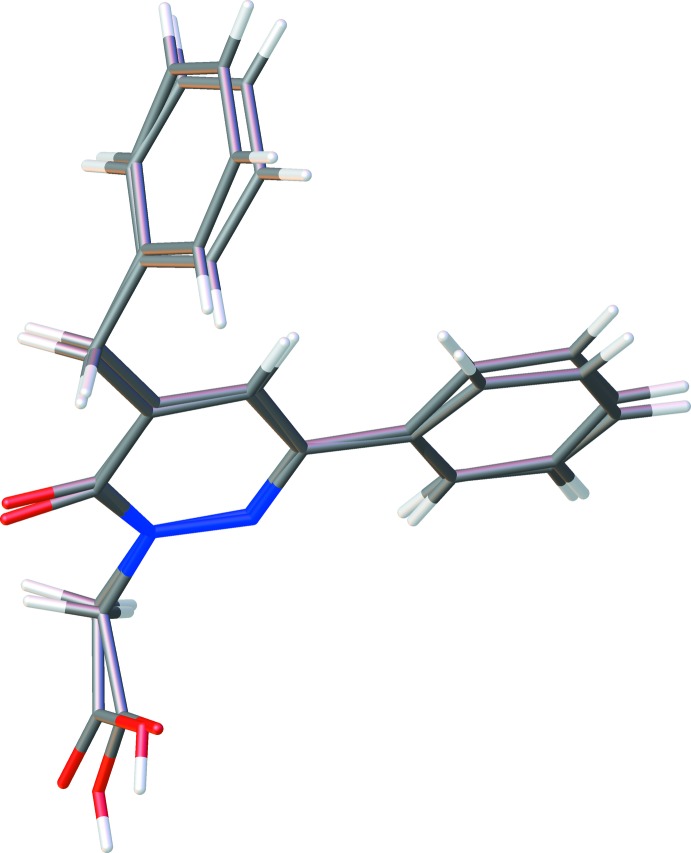
Structural overlap of mol­ecules (**I**) and (**II**).

The title compound is dimorphic with two monoclinic polymorphs. The mol­ecular structure of polymorph (**I**) is shown in Fig. 1 ▸ and that of polymorph (**II**) in Fig. 2 ▸. The differences in the conformations of the two mol­ecules is shown in the structural overlap drawing (Fig. 3 ▸). The main difference between (**I**) and (**II**) pertains to the OH function of the carboxyl group, which is reversed in the two mol­ecules. All other conformational features are quite similar in the mol­ecules of the two polymorphs. In (**I**), the phenyl ring (C1–C6) and the pyridazine ring (N1/N2/C10–C7) are nearly co-planar, making a dihedral angle of 5.92 (2)° whereas the phenyl ring of the benzyl group (C14–C19) is perpendicular to the pyridazine ring, with a dihedral angle of 89.91 (1)° (Fig. 1 ▸). In (**II**), the corresponding values are 15.44 (2) and 89.13 (1)°, respectively. In the mol­ecule of (**I**), the carboxyl group has a C12—O2 bond length of 1.277 (2) Å between the C atom and the OH function, and the C12=O3 bond length of the carbonyl group is 1.187 (2) Å. The corresponding values in (**II**) are 1.3057 (16) and 1.2108 (18) Å. The differences in the bond lengths of the two carb­oxy­lic groups can be attributed to their different roles in inter­molecular hydrogen bonding (see below). In both mol­ecules, weak intra­molecular hydrogen bonds [C—H⋯N for (**I**) and C—H⋯O for (**II**); Figs. 1 ▸ and 2 ▸, Tables 1 ▸ and 2 ▸] stabilize the mol­ecular conformation.

## Supra­molecular features   

**Figure 4:**
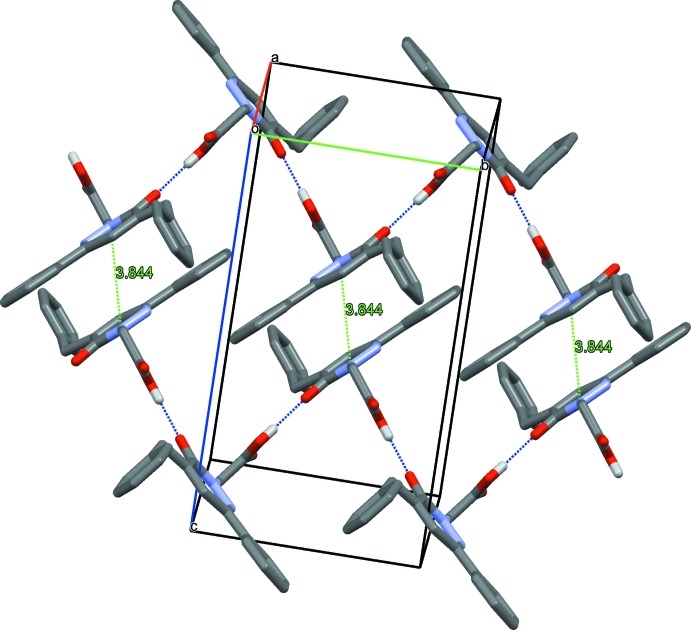
The crystal packing of (**I**). The O—H⋯O hydrogen bonds are shown as blue dotteded lines, and π–π contacts are represented by green dotted lines. For clarity, only H atoms involved in hydrogen bonding (white sticks) were included.

**Figure 5:**
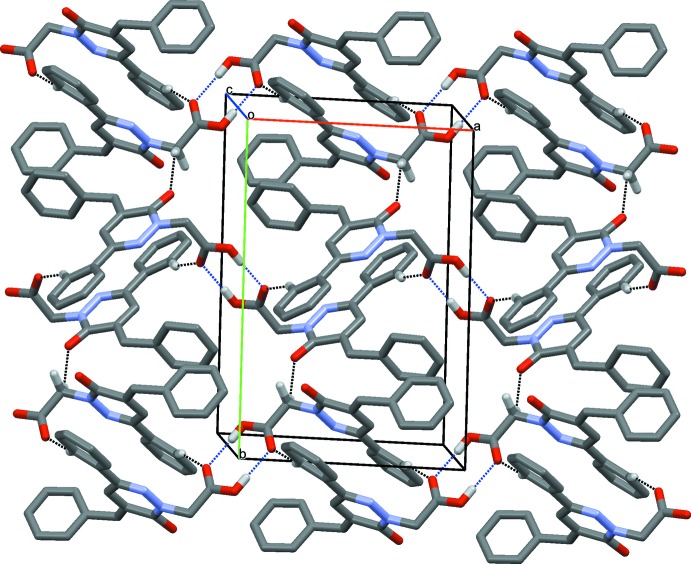
The crystal packing of (**II**), with O—H⋯O and C—H⋯O inter­actions shown as blue and black dotted lines, respectively.

In the crystal structure of (**I**), mol­ecules are linked by O2—H2⋯O1^i^ hydrogen bonds between the carb­oxy­lic OH function and the pyridazinone carbonyl O1 atom of a neighbouring mol­ecule, generating *C*(7) chains extending parallel to the *b-*axis direction (Fig. 4 ▸, Table 1 ▸). A weak π–π stacking inter­action occurs between the pyridazinone rings of inversion-related mol­ecules [*Cg*1⋯*Cg*1(1 − x, 1 − y,1 − z)], with a centroid–to–centroid distance of 3.8437 (12) Å and a slippage of 1.690 (*Cg*1 is the centroid of the N1/N2/C10–C7 ring) (Fig. 4 ▸). As a result of the reversed orientation of the carb­oxy­lic hy­droxy function, in the crystal structure of (**II**) the hydrogen-bonding scheme is different. Here mol­ecules are linked by pairs of O3—H3⋯O2^i^ hydrogen bonds between the carb­oxy­lic groups of neighbouring mol­ecules, forming inversion dimers with an 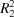
(8) ring motif. The dimers are linked by weak C5—H5⋯O2^ii^ and C11—H11*A*⋯O1^iii^ hydrogen bonds, forming *C*(8) chains extending parallel to the *b-*axis direction (Table 2 ▸, Fig. 5 ▸). The crystal packing of (**II**) also features weak π–π inter­actions involving the centroids of the N1/N2/C7–C10 (*Cg*1) and C14–C19 (*Cg*3) rings, with *Cg*1⋯*Cg*3(*x*, 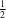 − *y*, −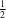 + *z*) = 4.3830 (12) Å.

## Database survey   

A search of the Cambridge Structural Database (CSD, version 5.40, update August 2019; Groom *et al.*, 2016 ▸) using 2-[6-oxopyridazin-1(6*H*)-yl]acetic acid as the main skeleton revealed the presence of three structures similar to the title compound, but with different substituents on the pyridazione ring, *viz*. ethyl 2-[6-oxo-3,4-diphenyl-1,6-di­hydro­pyridazin-1-yl]acetic acid acetate (CIPTOL; Aydın *et al.*, 2007 ▸), ethyl 3-methyl-6-oxo-5-[3-(tri­fluoro­meth­yl)phen­yl]-1,6-di­hydro-1-pyridazine­acetate (QANVOR; Xu *et al.*, 2005 ▸) and ethyl {4-[(5-chloro-1-benzo­furan-2-yl)meth­yl]-3-methyl-6-oxopyrida­zin-1(6*H*)-yl}acetate (XULSEE; Boukharsa *et al.*, 2015 ▸). Like in (**I**) and (**II**), the packing within the crystal structures of these compounds is dominated by O—H⋯O hydrogen bonds and consolidated by C—H⋯O inter­actions. In CIPTOL, the pyridazinone ring and two phenyl rings are inclined to each other by 72.73 (11) and 49.97 (10)° compared to the corres­ponding dihedral angles of 5.92 (2), 89.91 (1) and 15.44 (2)°, 89.13 (1)° in (**I**) and (**II**), respectively. In QANVOR, the 3-(tri­fluoro­meth­yl)phenyl and pyridazinone rings are approximately coplanar with a dihedral angle of 4.84 (13)°. In XULSEE, the dihedral angle between the benzo­furan ring system [maximum deviation 0.014 (2) Å] and the pyridazinone ring is 73.33 (8)°.

## Hirshfeld surface analysis   

**Figure 6:**
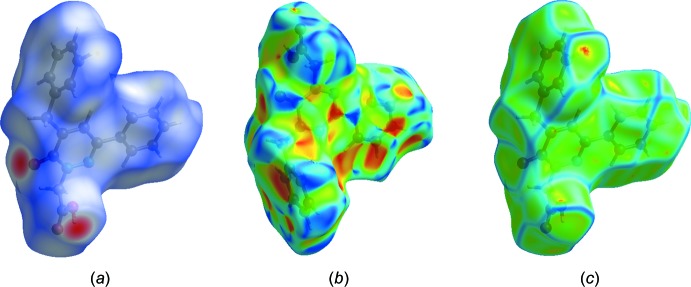
(*a*) The Hirshfeld surface of (**I**) mapped over *d*_norm_, and plotted in the range −0.7266 (red) to 1.4843 (blue) a.u.; (*b*) the Hirshfeld surface mapped over shape-index; (*c*) the Hirshfeld surface mapped over curvedness.

**Figure 7:**
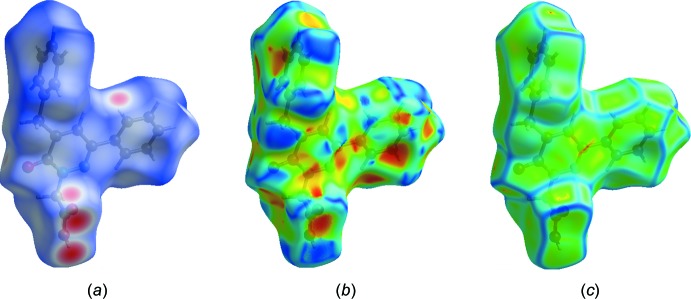
(*a*) The Hirshfeld surface of (**II**) mapped over *d*_norm_, and plotted in the range −0.7232 (red) to 1.3047 (blue) a.u.; (*b*) the Hirshfeld surface mapped over shape-index, (*c*) the Hirshfeld surface mapped over curvedness.

**Figure 8:**
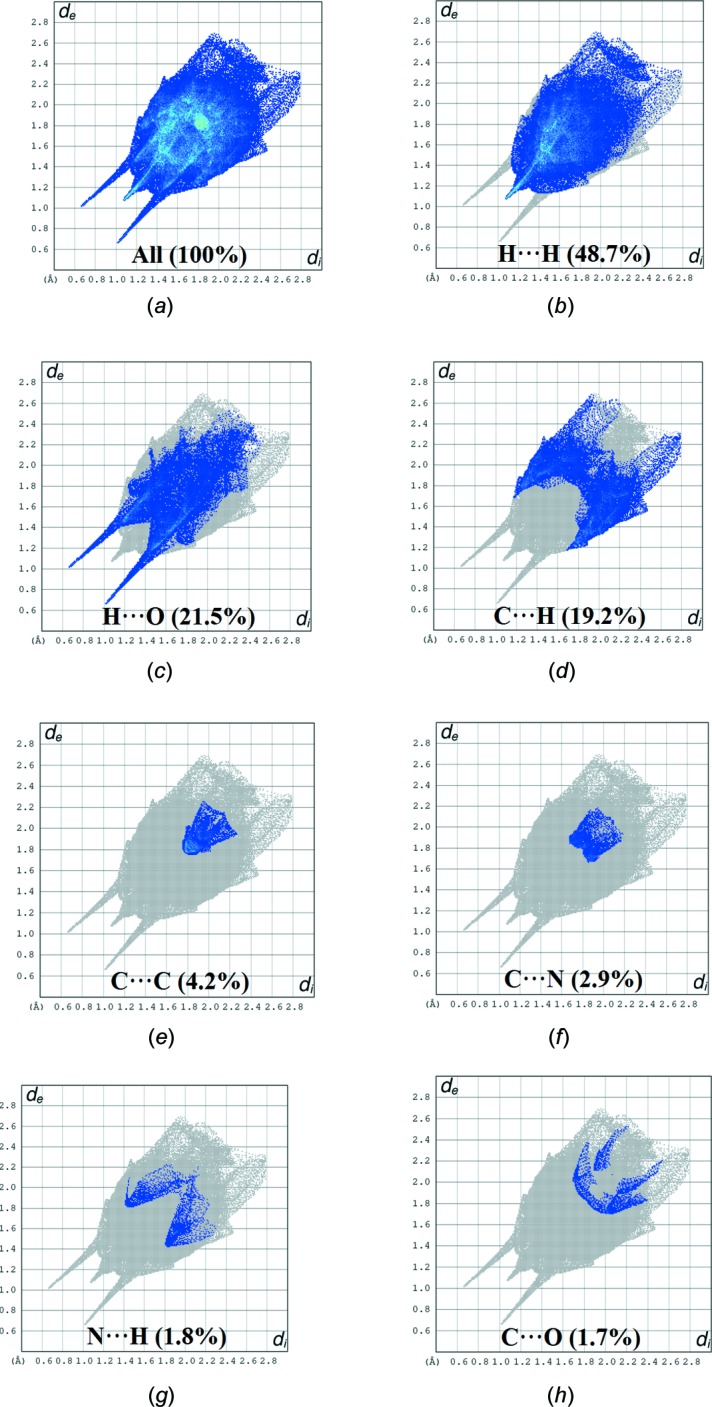
Two-dimensional fingerprint plots for (**I**): (*a*) all inter­molecular inter­actions; (*b*) H⋯H contacts; (*c*) H⋯O contacts; (*d*) C⋯H contacts; (*e*) C⋯C contacts; (*f*) C⋯N contacts; (*g*) N⋯H contacts; (*h*) C⋯O contacts.

**Figure 9:**
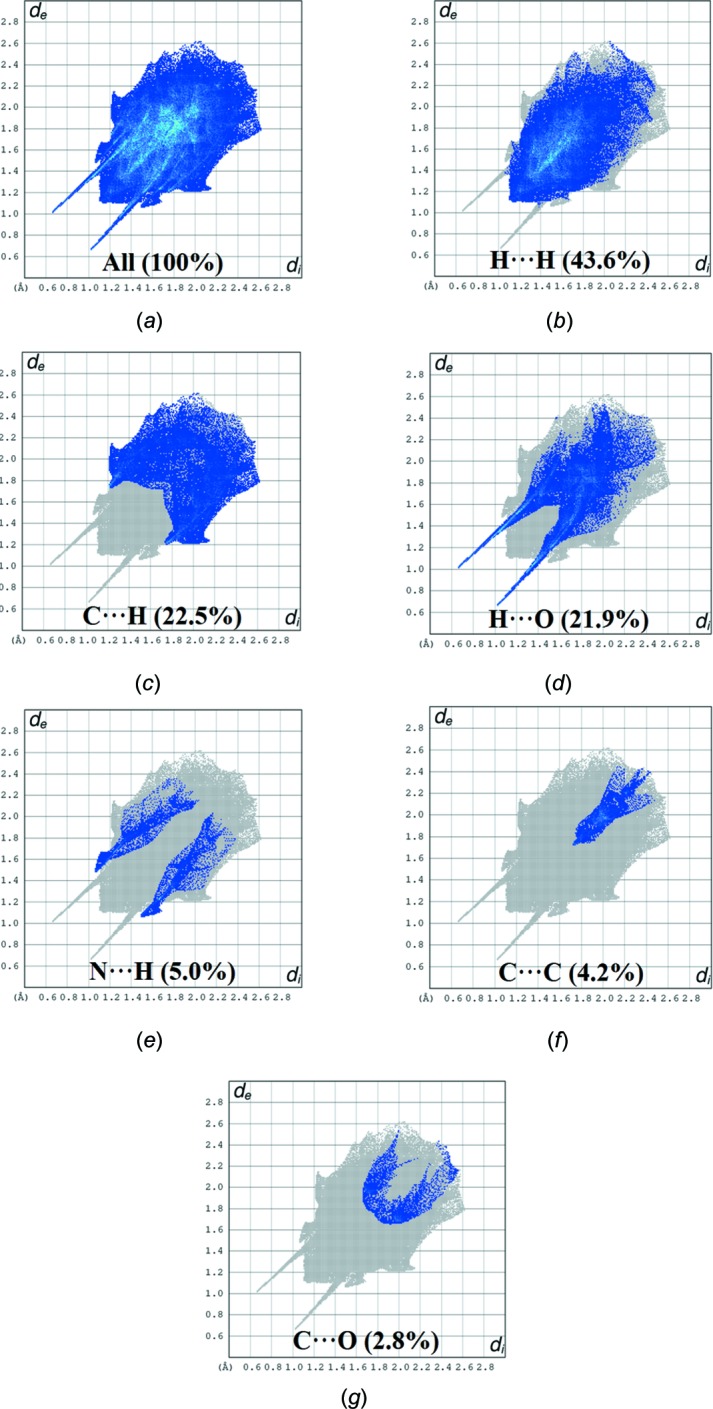
Two-dimensional fingerprint plots for (**II**): (*a*) all inter­molecular inter­actions; (*b*) H⋯H contacts; (*c*) C⋯H contacts; (*d*) H⋯O contacts; (*e*) N⋯H contacts; (*f*) C⋯C contacts; (*g*) C⋯O contacts.

Hirshfeld surface analysis was applied to qu­antify the inter­molecular contacts in (**I**) and (**II**), using *CrystalExplorer17.5* (Turner *et al.*, 2017 ▸). A standard (high) surface resolution with the three-dimensional *d*_norm_ surfaces plotted over a fixed colour scale of −0.7266 (red) to 1.4843 (blue) a.u. was used for (**I**) and of −0.7232 (red) to 1.3047 (blue) a.u. for (**II**). The bright-red spots on the Hirshfeld surface mapped over *d*_norm_ show the presence of O—H⋯O inter­actions with neighbouring mol­ecules in (**I**) (Fig. 6 ▸ *a*) and (**II**) (Fig. 7 ▸ *a*), respectively. The presence of red and blue triangles on the shape-index map [Fig. 6 ▸ *b* (**I**) and 7*b* (**II**)] are indicative for the presence of π–π stacking inter­actions. The curvedness plots show flat surface patches characteristic of planar stacking (Fig. 6 ▸ *c* and 7*c*). The complete two-dimensional fingerprint plots are shown in Fig. 8 ▸ *a* and 9*a* for (**I**) and (**II**). The H⋯H, H⋯O, C⋯H, C⋯C, C⋯N, N⋯H and C⋯O inter­actions are illustrated in Fig. 8 ▸ *b*–*h* for (**I**), and H⋯H, C⋯H, H⋯O, N⋯H, C⋯C and C⋯O inter­actions are illustrated in Fig. 9 ▸ *b*–*g* for (**II**). In both crystal structures, H⋯H inter­actions make the largest contributions to the overall Hirshfeld surfaces [48.7% for (**I**) and 43.6% for (**II**)]. As expected from the inter­molecular O—H⋯O and C—H⋯O contacts detailed in Tables 1 ▸ and 2 ▸, H⋯O contacts also account for a high percentage contributions [21.5% (**I**) and 21.9% (**II**)] and are indicated by a pair of wings at *d*_e_ + *d*_i_ ∼1.7 Å [Fig. 8 ▸ *c* (**I**) and 9*d* (**II**)]. The C⋯H contacts,with percentage contributions of 19.2% in (**I**) and 22.5% in (**II**) appear in the fingerprint plots as two distinct spikes at *d*_e_ + *d*_i_ ∼2.9 Å in (**I**) and 3.0 Å in (**II**) (Fig. 8 ▸ *d* and 9*c*). The C⋯C contacts, which refer to π–π inter­actions, contribute 4.2% of the Hirshfeld surfaces for both (**I**) and (**II**) (Fig. 8 ▸ *e* and 9*f*). There are additional N⋯H (5.0%) and C⋯O (2.8%) contacts in (**II**), while in (**I**) (where N⋯H = 1.8% and C⋯O = 1.7%), C⋯N (2.9%) inter­actions are also observed.

## Synthesis and crystallization   

A suspension of ethyl 2-(5-benzyl-6-oxo-3-phenyl­pyridazin-1(6*H*)-yl)acetate (3.6 mmol), and 6 *N* NaOH (14.4 mmol) in ethanol (50 ml) was stirred at 353 K for 4 h. The mixture was then concentrated *in vacuo*, diluted with cold water, and acidified with 6 *N* HCl. The final product was filtered off by suction filtration and recrystallized from ethanol or methanol. Single crystals of (**I**) were obtained by slow evaporation of an ethano­lic solution at room temperature, and single crystals of (**II**) were obtained by slow evaporation of a methano­lic solution at room temperature.

## Refinement   

Crystal data, data collection and structure refinement details are summarized in Table 3 ▸. The atom labelling for mol­ecules of (**I**) and (**II**) is identical. In the refinement of (**I**), SIMU, DELU and ISOR commands were used for atoms C12 and O3. For both structures, hydrogen atoms of the carb­oxy­lic group were located in a difference-Fourier map and were refined with a fixed O—H distance of 0.82 Å and with *U*_iso_(H) = 1.5*U*_eq_(O). All other hydrogen atoms were placed in calculated positions, with C—H = 0.93–0.96 Å and allowed to ride on their parent atoms with *U*_iso_(H) = 1.5*U*_eq_(C-meth­yl) and 1.2*U*_eq_(C) for other H atoms.

## Supplementary Material

Crystal structure: contains datablock(s) I, II. DOI: 10.1107/S2056989020002406/wm5541sup1.cif

Structure factors: contains datablock(s) I. DOI: 10.1107/S2056989020002406/wm5541Isup2.hkl

Structure factors: contains datablock(s) II. DOI: 10.1107/S2056989020002406/wm5541IIsup3.hkl

Supporting information file. DOI: 10.1107/S2056989020002406/wm5541Isup4.cml

CCDC references: 1985197, 1985196

Additional supporting information:  crystallographic information; 3D view; checkCIF report

## Figures and Tables

**Table 1:** Hydrogen-bond geometry (Å, °) for **I** [Chem scheme1]

*D*—H⋯*A*	*D*—H	H⋯*A*	*D*⋯*A*	*D*—H⋯*A*
O2—H2⋯O1^i^	0.82	1.82	2.593 (2)	156
C1—H1⋯N1	0.93	2.47	2.780 (3)	100

Symmetry code: (i) 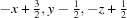.

**Table 2:** Hydrogen-bond geometry (Å, °) for **II** [Chem scheme1]

*D*—H⋯*A*	*D*—H	H⋯*A*	*D*⋯*A*	*D*—H⋯*A*
C11—H11*B*⋯O1	0.97	2.39	2.7325 (19)	100
O2—H3⋯O3^i^	0.82	1.84	2.6599 (16)	177
C5—H5⋯O3^ii^	0.93	2.40	3.280 (2)	159
C11—H11*A*⋯O1^iii^	0.97	2.47	3.2814 (19)	141

Symmetry codes: (i) 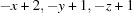; (ii) 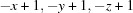; (iii) 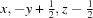.

**Table 3:** Experimental details

	**I**	**II**
Crystal data		
Chemical formula	C_19_H_16_N_2_O_3_	C_19_H_16_N_2_O_3_
*M*_r_	320.34	320.34
Crystal system, space group	Monoclinic, *P*2_1_/*n*	Monoclinic, *P*2_1_/*c*
Temperature (K)	296	296
*a*, *b*, *c* (Å)	10.5500 (8), 9.3679 (6), 16.5606 (15)	10.5976 (6), 15.5500 (7), 10.3731 (7)
β (°)	93.886 (7)	109.120 (5)
*V* (Å^3^)	1632.9 (2)	1615.11 (17)
*Z*	4	4
Radiation type	Mo *K*α	Mo *K*α
μ (mm^−1^)	0.09	0.09
Crystal size (mm)	0.58 × 0.43 × 0.34	0.77 × 0.70 × 0.59

Data collection		
Diffractometer	Stoe IPDS 2	STOE IPDS 2
Absorption correction	Integration (*X-RED32*; Stoe & Cie, 2002 ▸)	Integration (*X-RED32*; Stoe & Cie, 2002 ▸)
*T*_min_, *T*_max_	0.961, 0.981	0.950, 0.966
No. of measured, independent and observed [*I* > 2σ(*I*)] reflections	12987, 4603, 1989	12114, 4562, 2560
*R*_int_	0.039	0.037
(sin θ/λ)_max_ (Å^−1^)	0.698	0.699

Refinement		
*R*[*F* ^2^ > 2σ(*F* ^2^)], *wR*(*F* ^2^), *S*	0.053, 0.158, 0.89	0.049, 0.131, 0.98
No. of reflections	4603	4562
No. of parameters	217	218
No. of restraints	19	0
H-atom treatment	H-atom parameters constrained	H-atom parameters constrained
Δρ_max_, Δρ_min_ (e Å^−3^)	0.35, −0.34	0.21, −0.21

Computer programs: *X-AREA* and *X-RED32* (Stoe & Cie, 2002 ▸), *SHELXT2017/1* (Sheldrick, 2015*a* ▸), *SHELXL2018/3* (Sheldrick, 2015*b* ▸), *Mercury* (Macrae *et al.*, 2020 ▸), *WinGX* (Farrugia, 2012 ▸), *PLATON* (Spek, 2020 ▸) and *publCIF* (Westrip, 2010 ▸).

## supplementary crystallographic information

### 2-(5-Benzyl-6-oxo-3-phenyl-1,6-dihydropyridazin-1-yl)acetic acid (I) Crystal data

**Table:**

C_19_H_16_N_2_O_3_	*F*(000) = 672
*M**_r_* = 320.34	*D*_x_ = 1.303 Mg m^−^^3^
Monoclinic, *P*2_1_/*n*	Mo *K*α radiation, λ = 0.71073 Å
*a* = 10.5500 (8) Å	Cell parameters from 9543 reflections
*b* = 9.3679 (6) Å	θ = 1.9–29.8°
*c* = 16.5606 (15) Å	µ = 0.09 mm^−^^1^
β = 93.886 (7)°	*T* = 296 K
*V* = 1632.9 (2) Å^3^	Prism, colorless
*Z* = 4	0.58 × 0.43 × 0.34 mm

### 2-(5-Benzyl-6-oxo-3-phenyl-1,6-dihydropyridazin-1-yl)acetic acid (I) Data collection

**Table:**

Stoe IPDS 2 diffractometer	4603 independent reflections
Radiation source: sealed X-ray tube, 12 x 0.4 mm long-fine focus	1989 reflections with *I* > 2σ(*I*)
Plane graphite monochromator	*R*_int_ = 0.039
Detector resolution: 6.67 pixels mm^-1^	θ_max_ = 29.7°, θ_min_ = 2.4°
rotation method scans	*h* = −12→14
Absorption correction: integration (X-RED32; Stoe & Cie, 2002)	*k* = −13→12
*T*_min_ = 0.961, *T*_max_ = 0.981	*l* = −23→23
12987 measured reflections	

### 2-(5-Benzyl-6-oxo-3-phenyl-1,6-dihydropyridazin-1-yl)acetic acid (I) Refinement

**Table:**

Refinement on *F*^2^	19 restraints
Least-squares matrix: full	Hydrogen site location: inferred from neighbouring sites
*R*[*F*^2^ > 2σ(*F*^2^)] = 0.053	H-atom parameters constrained
*wR*(*F*^2^) = 0.158	*w* = 1/[σ^2^(*F*_o_^2^) + (0.0772*P*)^2^] where *P* = (*F*_o_^2^ + 2*F*_c_^2^)/3
*S* = 0.89	(Δ/σ)_max_ < 0.001
4603 reflections	Δρ_max_ = 0.35 e Å^−^^3^
217 parameters	Δρ_min_ = −0.34 e Å^−^^3^

### 2-(5-Benzyl-6-oxo-3-phenyl-1,6-dihydropyridazin-1-yl)acetic acid (I) Special details

**Table:**

Geometry. All esds (except the esd in the dihedral angle between two l.s. planes) are estimated using the full covariance matrix. The cell esds are taken into account individually in the estimation of esds in distances, angles and torsion angles; correlations between esds in cell parameters are only used when they are defined by crystal symmetry. An approximate (isotropic) treatment of cell esds is used for estimating esds involving l.s. planes.

### 2-(5-Benzyl-6-oxo-3-phenyl-1,6-dihydropyridazin-1-yl)acetic acid (I) Fractional atomic coordinates and isotropic or equivalent isotropic displacement parameters (Å^2^)

**Table:**

	*x*	*y*	*z*	*U*_iso_*/*U*_eq_	
N2	0.63345 (14)	0.43785 (17)	0.40115 (9)	0.0550 (4)	
N1	0.56137 (14)	0.34515 (16)	0.44077 (9)	0.0539 (4)	
O1	0.66753 (13)	0.60906 (17)	0.31011 (10)	0.0811 (5)	
O2	0.76216 (13)	0.27628 (19)	0.30333 (10)	0.0914 (6)	
H2	0.804328	0.226747	0.274357	0.137*	
C7	0.43814 (16)	0.35514 (19)	0.42800 (10)	0.0508 (4)	
C6	0.36108 (18)	0.2513 (2)	0.47105 (11)	0.0536 (4)	
C8	0.38202 (17)	0.4630 (2)	0.37706 (11)	0.0567 (5)	
H8	0.294010	0.471278	0.371699	0.068*	
C10	0.58990 (18)	0.5384 (2)	0.34591 (11)	0.0589 (5)	
C12	0.83446 (19)	0.3288 (2)	0.36076 (12)	0.0612 (5)	
C9	0.45351 (17)	0.5535 (2)	0.33636 (11)	0.0594 (5)	
O3	0.94446 (16)	0.3027 (3)	0.37082 (12)	0.1213 (7)	
C11	0.77005 (17)	0.4220 (2)	0.41864 (12)	0.0634 (5)	
H11A	0.785002	0.382843	0.472680	0.076*	
H11B	0.808847	0.515829	0.418553	0.076*	
C14	0.26299 (19)	0.7017 (2)	0.29161 (12)	0.0638 (5)	
C5	0.2306 (2)	0.2435 (2)	0.45719 (13)	0.0677 (6)	
H5	0.189656	0.304979	0.419821	0.081*	
C15	0.2264 (2)	0.7924 (3)	0.35035 (15)	0.0794 (7)	
H15	0.288323	0.838285	0.383550	0.095*	
C4	0.1600 (2)	0.1465 (3)	0.49763 (14)	0.0780 (6)	
H4	0.072279	0.143929	0.487252	0.094*	
C13	0.4010 (2)	0.6709 (3)	0.28207 (15)	0.0822 (7)	
H13A	0.411644	0.644798	0.226260	0.099*	
H13B	0.449726	0.757252	0.293614	0.099*	
C17	0.0092 (2)	0.7521 (3)	0.31546 (16)	0.0888 (8)	
H17	−0.075763	0.769037	0.323863	0.107*	
C19	0.1676 (2)	0.6361 (3)	0.24455 (14)	0.0836 (7)	
H19	0.188376	0.573613	0.203863	0.100*	
C3	0.2156 (3)	0.0557 (3)	0.55173 (15)	0.0859 (7)	
H3	0.167270	−0.008738	0.579307	0.103*	
C1	0.4167 (2)	0.1565 (3)	0.52585 (16)	0.0904 (8)	
H1	0.504386	0.157532	0.536391	0.109*	
C16	0.1008 (2)	0.8173 (3)	0.36152 (17)	0.0958 (8)	
H16	0.078911	0.880366	0.401664	0.115*	
C18	0.0408 (2)	0.6619 (3)	0.25690 (16)	0.0901 (8)	
H18	−0.022580	0.616712	0.224545	0.108*	
C2	0.3444 (3)	0.0599 (3)	0.56547 (18)	0.1078 (10)	
H2A	0.384238	−0.003519	0.602205	0.129*	

### 2-(5-Benzyl-6-oxo-3-phenyl-1,6-dihydropyridazin-1-yl)acetic acid (I) Atomic displacement parameters (Å^2^)

**Table:**

	*U*^11^	*U*^22^	*U*^33^	*U*^12^	*U*^13^	*U*^23^
N2	0.0449 (8)	0.0597 (10)	0.0611 (9)	0.0062 (7)	0.0087 (7)	−0.0024 (8)
N1	0.0496 (9)	0.0560 (9)	0.0565 (8)	0.0062 (7)	0.0063 (7)	−0.0028 (7)
O1	0.0572 (9)	0.0901 (11)	0.0988 (11)	0.0021 (8)	0.0255 (8)	0.0262 (9)
O2	0.0511 (8)	0.1258 (14)	0.0963 (11)	0.0206 (9)	−0.0021 (8)	−0.0447 (10)
C7	0.0487 (10)	0.0515 (11)	0.0526 (9)	0.0073 (8)	0.0051 (8)	−0.0036 (8)
C6	0.0549 (11)	0.0523 (11)	0.0542 (10)	0.0049 (9)	0.0079 (8)	−0.0018 (8)
C8	0.0457 (10)	0.0611 (11)	0.0643 (11)	0.0097 (9)	0.0113 (8)	0.0068 (9)
C10	0.0512 (10)	0.0611 (12)	0.0662 (11)	0.0069 (10)	0.0176 (9)	0.0038 (10)
C12	0.0514 (7)	0.0670 (9)	0.0653 (8)	0.0051 (7)	0.0037 (7)	0.0008 (7)
C9	0.0503 (11)	0.0658 (12)	0.0638 (11)	0.0123 (9)	0.0163 (9)	0.0119 (9)
O3	0.0569 (8)	0.1817 (15)	0.1240 (12)	0.0325 (10)	−0.0028 (8)	−0.0424 (12)
C11	0.0459 (11)	0.0735 (14)	0.0701 (12)	0.0039 (9)	−0.0017 (9)	−0.0047 (10)
C14	0.0580 (12)	0.0676 (13)	0.0666 (12)	0.0121 (10)	0.0103 (10)	0.0237 (10)
C5	0.0596 (12)	0.0704 (14)	0.0739 (13)	0.0014 (10)	0.0100 (10)	0.0141 (10)
C15	0.0572 (13)	0.0833 (16)	0.0964 (16)	0.0031 (12)	−0.0039 (11)	−0.0097 (13)
C4	0.0619 (13)	0.0798 (15)	0.0939 (16)	−0.0060 (12)	0.0162 (11)	0.0109 (13)
C13	0.0623 (13)	0.0909 (17)	0.0962 (16)	0.0209 (12)	0.0249 (11)	0.0393 (14)
C17	0.0555 (13)	0.119 (2)	0.0923 (17)	0.0121 (14)	0.0047 (12)	−0.0046 (16)
C19	0.0876 (18)	0.0948 (18)	0.0684 (13)	0.0214 (14)	0.0051 (12)	−0.0045 (13)
C3	0.0887 (18)	0.0720 (15)	0.0992 (17)	−0.0067 (14)	0.0234 (14)	0.0203 (13)
C1	0.0660 (14)	0.0925 (18)	0.1114 (19)	0.0008 (13)	−0.0045 (13)	0.0438 (16)
C16	0.0666 (15)	0.118 (2)	0.1027 (18)	0.0164 (15)	0.0038 (13)	−0.0330 (17)
C18	0.0709 (16)	0.112 (2)	0.0846 (16)	−0.0008 (15)	−0.0154 (12)	−0.0093 (15)
C2	0.100 (2)	0.100 (2)	0.122 (2)	0.0027 (17)	−0.0042 (17)	0.0588 (18)

### 2-(5-Benzyl-6-oxo-3-phenyl-1,6-dihydropyridazin-1-yl)acetic acid (I) Geometric parameters (Å, º)

**Table:**

N2—N1	1.353 (2)	C14—C13	1.503 (3)
N2—C10	1.371 (2)	C5—C4	1.377 (3)
N2—C11	1.458 (2)	C5—H5	0.9300
N1—C7	1.306 (2)	C15—C16	1.371 (3)
O1—C10	1.235 (2)	C15—H15	0.9300
O2—C12	1.277 (2)	C4—C3	1.341 (3)
O2—H2	0.8200	C4—H4	0.9300
C7—C8	1.420 (2)	C13—H13A	0.9700
C7—C6	1.481 (3)	C13—H13B	0.9700
C6—C1	1.373 (3)	C17—C16	1.337 (3)
C6—C5	1.382 (3)	C17—C18	1.345 (4)
C8—C9	1.346 (3)	C17—H17	0.9300
C8—H8	0.9300	C19—C18	1.389 (3)
C10—C9	1.444 (3)	C19—H19	0.9300
C12—O3	1.187 (2)	C3—C2	1.363 (4)
C12—C11	1.494 (3)	C3—H3	0.9300
C9—C13	1.502 (3)	C1—C2	1.378 (4)
C11—H11A	0.9700	C1—H1	0.9300
C11—H11B	0.9700	C16—H16	0.9300
C14—C15	1.367 (3)	C18—H18	0.9300
C14—C19	1.375 (3)	C2—H2A	0.9300
			
N1—N2—C10	126.22 (15)	C6—C5—H5	119.3
N1—N2—C11	114.74 (15)	C14—C15—C16	121.6 (2)
C10—N2—C11	119.00 (16)	C14—C15—H15	119.2
C7—N1—N2	117.48 (15)	C16—C15—H15	119.2
C12—O2—H2	109.5	C3—C4—C5	121.1 (2)
N1—C7—C8	121.17 (17)	C3—C4—H4	119.4
N1—C7—C6	116.60 (16)	C5—C4—H4	119.4
C8—C7—C6	122.21 (16)	C9—C13—C14	113.43 (17)
C1—C6—C5	116.86 (19)	C9—C13—H13A	108.9
C1—C6—C7	121.27 (18)	C14—C13—H13A	108.9
C5—C6—C7	121.86 (17)	C9—C13—H13B	108.9
C9—C8—C7	121.38 (17)	C14—C13—H13B	108.9
C9—C8—H8	119.3	H13A—C13—H13B	107.7
C7—C8—H8	119.3	C16—C17—C18	119.6 (2)
O1—C10—N2	119.04 (17)	C16—C17—H17	120.2
O1—C10—C9	125.67 (19)	C18—C17—H17	120.2
N2—C10—C9	115.28 (16)	C14—C19—C18	120.9 (2)
O3—C12—O2	123.7 (2)	C14—C19—H19	119.5
O3—C12—C11	120.9 (2)	C18—C19—H19	119.5
O2—C12—C11	115.36 (17)	C4—C3—C2	118.6 (2)
C8—C9—C10	118.14 (18)	C4—C3—H3	120.7
C8—C9—C13	124.38 (17)	C2—C3—H3	120.7
C10—C9—C13	117.48 (17)	C6—C1—C2	121.0 (2)
N2—C11—C12	114.75 (16)	C6—C1—H1	119.5
N2—C11—H11A	108.6	C2—C1—H1	119.5
C12—C11—H11A	108.6	C17—C16—C15	120.9 (2)
N2—C11—H11B	108.6	C17—C16—H16	119.6
C12—C11—H11B	108.6	C15—C16—H16	119.6
H11A—C11—H11B	107.6	C17—C18—C19	120.3 (2)
C15—C14—C19	116.8 (2)	C17—C18—H18	119.9
C15—C14—C13	121.1 (2)	C19—C18—H18	119.9
C19—C14—C13	122.1 (2)	C3—C2—C1	121.1 (2)
C4—C5—C6	121.3 (2)	C3—C2—H2A	119.4
C4—C5—H5	119.3	C1—C2—H2A	119.4

### 2-(5-Benzyl-6-oxo-3-phenyl-1,6-dihydropyridazin-1-yl)acetic acid (I) Hydrogen-bond geometry (Å, º)

**Table:**

*D*—H···*A*	*D*—H	H···*A*	*D*···*A*	*D*—H···*A*
O2—H2···O1^i^	0.82	1.82	2.593 (2)	156
C1—H1···N1	0.93	2.47	2.780 (3)	100

Symmetry code: (i) −*x*+3/2, *y*−1/2, −*z*+1/2.

### (II) Crystal data

**Table:**

C_19_H_16_N_2_O_3_	*F*(000) = 672
*M**_r_* = 320.34	*D*_x_ = 1.317 Mg m^−^^3^
Monoclinic, *P*2_1_/*c*	Mo *K*α radiation, λ = 0.71073 Å
*a* = 10.5976 (6) Å	Cell parameters from 11065 reflections
*b* = 15.5500 (7) Å	θ = 2.0–30.2°
*c* = 10.3731 (7) Å	µ = 0.09 mm^−^^1^
β = 109.120 (5)°	*T* = 296 K
*V* = 1615.11 (17) Å^3^	Prism, colorless
*Z* = 4	0.77 × 0.70 × 0.59 mm

### (II) Data collection

**Table:**

STOE IPDS 2 diffractometer	4562 independent reflections
Radiation source: sealed X-ray tube, 12 x 0.4 mm long-fine focus	2560 reflections with *I* > 2σ(*I*)
Plane graphite monochromator	*R*_int_ = 0.037
Detector resolution: 6.67 pixels mm^-1^	θ_max_ = 29.8°, θ_min_ = 2.0°
rotation method scans	*h* = −14→14
Absorption correction: integration (X-RED32; Stoe & Cie, 2002)	*k* = −21→21
*T*_min_ = 0.950, *T*_max_ = 0.966	*l* = −9→14
12114 measured reflections	

### (II) Refinement

**Table:**

Refinement on *F*^2^	0 restraints
Least-squares matrix: full	Hydrogen site location: inferred from neighbouring sites
*R*[*F*^2^ > 2σ(*F*^2^)] = 0.049	H-atom parameters constrained
*wR*(*F*^2^) = 0.131	*w* = 1/[σ^2^(*F*_o_^2^) + (0.0658*P*)^2^] where *P* = (*F*_o_^2^ + 2*F*_c_^2^)/3
*S* = 0.98	(Δ/σ)_max_ < 0.001
4562 reflections	Δρ_max_ = 0.21 e Å^−^^3^
218 parameters	Δρ_min_ = −0.21 e Å^−^^3^

### (II) Special details

**Table:**

Geometry. All esds (except the esd in the dihedral angle between two l.s. planes) are estimated using the full covariance matrix. The cell esds are taken into account individually in the estimation of esds in distances, angles and torsion angles; correlations between esds in cell parameters are only used when they are defined by crystal symmetry. An approximate (isotropic) treatment of cell esds is used for estimating esds involving l.s. planes.

### (II) Fractional atomic coordinates and isotropic or equivalent isotropic displacement parameters (Å^2^)

**Table:**

	*x*	*y*	*z*	*U*_iso_*/*U*_eq_	
O1	0.72419 (10)	0.27629 (8)	0.59311 (12)	0.0685 (3)	
O3	0.85719 (10)	0.46576 (8)	0.50173 (13)	0.0697 (3)	
O2	0.97177 (11)	0.41088 (9)	0.37548 (13)	0.0746 (4)	
H3	1.024031	0.449746	0.410547	0.112*	
N1	0.56525 (10)	0.40450 (8)	0.30448 (12)	0.0469 (3)	
N2	0.65255 (10)	0.35304 (8)	0.39742 (12)	0.0482 (3)	
C7	0.45128 (12)	0.42124 (9)	0.32229 (14)	0.0448 (3)	
C10	0.63572 (13)	0.31919 (10)	0.51338 (15)	0.0505 (3)	
C8	0.41953 (13)	0.38747 (10)	0.43611 (15)	0.0518 (3)	
H8	0.336938	0.399584	0.445235	0.062*	
C6	0.35817 (13)	0.47589 (9)	0.21629 (14)	0.0467 (3)	
C9	0.50740 (13)	0.33851 (10)	0.52997 (15)	0.0510 (3)	
C12	0.87184 (13)	0.41235 (11)	0.42288 (15)	0.0540 (4)	
C11	0.77702 (13)	0.33946 (10)	0.37033 (16)	0.0529 (4)	
H11A	0.758711	0.333700	0.272786	0.064*	
H11B	0.817891	0.286421	0.413433	0.064*	
C14	0.34689 (14)	0.30038 (11)	0.65991 (16)	0.0559 (4)	
C1	0.37838 (15)	0.48952 (12)	0.09292 (17)	0.0629 (4)	
H1	0.450530	0.463405	0.076387	0.076*	
C13	0.48712 (15)	0.29980 (13)	0.65443 (18)	0.0679 (5)	
H13A	0.543928	0.330261	0.733863	0.082*	
H13B	0.517681	0.240631	0.661889	0.082*	
C2	0.29390 (17)	0.54093 (13)	−0.00593 (19)	0.0744 (5)	
H2	0.309754	0.549564	−0.088011	0.089*	
C5	0.25091 (17)	0.51563 (13)	0.23724 (19)	0.0733 (5)	
H5	0.234983	0.508074	0.319510	0.088*	
C3	0.18788 (19)	0.57899 (13)	0.0157 (2)	0.0814 (6)	
H3A	0.129995	0.613227	−0.051425	0.098*	
C19	0.3040 (2)	0.36098 (15)	0.7325 (2)	0.0846 (6)	
H19	0.361300	0.405216	0.775869	0.102*	
C15	0.25885 (18)	0.23814 (14)	0.5959 (2)	0.0837 (6)	
H15	0.283752	0.197158	0.543528	0.100*	
C17	0.09285 (19)	0.29337 (18)	0.6803 (3)	0.0941 (7)	
H17	0.008047	0.290075	0.688145	0.113*	
C4	0.1669 (2)	0.56655 (15)	0.1373 (2)	0.0960 (7)	
H4	0.094447	0.592977	0.152843	0.115*	
C18	0.1777 (2)	0.35732 (18)	0.7422 (3)	0.1008 (7)	
H18	0.150476	0.399081	0.791625	0.121*	
C16	0.1334 (2)	0.23488 (17)	0.6074 (3)	0.1056 (8)	
H16	0.075399	0.191088	0.563708	0.127*	

### (II) Atomic displacement parameters (Å^2^)

**Table:**

	*U*^11^	*U*^22^	*U*^33^	*U*^12^	*U*^13^	*U*^23^
O1	0.0598 (6)	0.0850 (8)	0.0606 (7)	0.0170 (6)	0.0196 (5)	0.0211 (6)
O3	0.0610 (6)	0.0868 (8)	0.0744 (8)	−0.0176 (6)	0.0402 (6)	−0.0276 (6)
O2	0.0615 (6)	0.1001 (10)	0.0777 (8)	−0.0164 (6)	0.0437 (6)	−0.0276 (7)
N1	0.0482 (5)	0.0524 (7)	0.0415 (6)	0.0005 (5)	0.0167 (5)	0.0007 (5)
N2	0.0435 (5)	0.0561 (7)	0.0464 (7)	0.0002 (5)	0.0166 (5)	−0.0001 (5)
C7	0.0457 (6)	0.0494 (8)	0.0417 (7)	−0.0021 (6)	0.0175 (5)	−0.0003 (6)
C10	0.0493 (7)	0.0556 (9)	0.0462 (8)	−0.0019 (6)	0.0152 (6)	0.0019 (7)
C8	0.0466 (6)	0.0643 (9)	0.0485 (8)	0.0003 (6)	0.0212 (6)	0.0065 (7)
C6	0.0499 (7)	0.0503 (8)	0.0442 (7)	0.0014 (6)	0.0212 (6)	0.0016 (6)
C9	0.0495 (7)	0.0586 (9)	0.0467 (8)	−0.0039 (6)	0.0180 (6)	0.0058 (7)
C12	0.0459 (7)	0.0737 (10)	0.0471 (8)	0.0002 (7)	0.0218 (6)	−0.0021 (7)
C11	0.0467 (7)	0.0654 (10)	0.0497 (8)	0.0037 (6)	0.0200 (6)	−0.0034 (7)
C14	0.0561 (7)	0.0661 (10)	0.0481 (8)	0.0005 (7)	0.0207 (6)	0.0174 (7)
C1	0.0595 (8)	0.0819 (12)	0.0563 (9)	0.0189 (8)	0.0310 (7)	0.0177 (8)
C13	0.0581 (8)	0.0897 (13)	0.0592 (10)	0.0047 (8)	0.0236 (7)	0.0264 (9)
C2	0.0784 (10)	0.0946 (13)	0.0575 (10)	0.0234 (10)	0.0323 (8)	0.0246 (9)
C5	0.0790 (10)	0.0930 (13)	0.0618 (10)	0.0284 (9)	0.0419 (9)	0.0175 (9)
C3	0.0832 (11)	0.0951 (15)	0.0701 (12)	0.0376 (10)	0.0307 (9)	0.0296 (10)
C19	0.0799 (11)	0.0973 (15)	0.0831 (14)	−0.0166 (10)	0.0355 (10)	−0.0194 (11)
C15	0.0728 (11)	0.0753 (13)	0.1021 (16)	−0.0058 (10)	0.0273 (10)	−0.0127 (11)
C17	0.0596 (10)	0.138 (2)	0.0901 (16)	−0.0055 (13)	0.0324 (10)	0.0234 (15)
C4	0.0908 (12)	0.1224 (18)	0.0917 (15)	0.0572 (13)	0.0528 (11)	0.0360 (13)
C18	0.0848 (13)	0.140 (2)	0.0911 (16)	0.0060 (14)	0.0478 (12)	−0.0147 (15)
C16	0.0719 (12)	0.0996 (17)	0.138 (2)	−0.0280 (12)	0.0244 (13)	−0.0066 (16)

### (II) Geometric parameters (Å, º)

**Table:**

O1—C10	1.2249 (17)	C14—C13	1.506 (2)
O3—C12	1.2108 (18)	C1—C2	1.375 (2)
O2—C12	1.3057 (16)	C1—H1	0.9300
O2—H3	0.8200	C13—H13A	0.9700
N1—C7	1.3064 (16)	C13—H13B	0.9700
N1—N2	1.3570 (16)	C2—C3	1.352 (2)
N2—C10	1.3771 (18)	C2—H2	0.9300
N2—C11	1.4513 (17)	C5—C4	1.375 (3)
C7—C8	1.4294 (19)	C5—H5	0.9300
C7—C6	1.4813 (19)	C3—C4	1.364 (3)
C10—C9	1.4568 (19)	C3—H3A	0.9300
C8—C9	1.342 (2)	C19—C18	1.376 (3)
C8—H8	0.9300	C19—H19	0.9300
C6—C5	1.372 (2)	C15—C16	1.374 (3)
C6—C1	1.381 (2)	C15—H15	0.9300
C9—C13	1.502 (2)	C17—C16	1.340 (4)
C12—C11	1.494 (2)	C17—C18	1.354 (3)
C11—H11A	0.9700	C17—H17	0.9300
C11—H11B	0.9700	C4—H4	0.9300
C14—C15	1.357 (2)	C18—H18	0.9300
C14—C19	1.373 (3)	C16—H16	0.9300
			
C12—O2—H3	109.5	C6—C1—H1	119.4
C7—N1—N2	117.63 (11)	C9—C13—C14	116.80 (13)
N1—N2—C10	126.35 (11)	C9—C13—H13A	108.1
N1—N2—C11	113.56 (11)	C14—C13—H13A	108.1
C10—N2—C11	119.97 (12)	C9—C13—H13B	108.1
N1—C7—C8	121.48 (12)	C14—C13—H13B	108.1
N1—C7—C6	115.55 (12)	H13A—C13—H13B	107.3
C8—C7—C6	122.96 (11)	C3—C2—C1	120.27 (16)
O1—C10—N2	120.56 (12)	C3—C2—H2	119.9
O1—C10—C9	124.76 (13)	C1—C2—H2	119.9
N2—C10—C9	114.67 (12)	C6—C5—C4	120.37 (16)
C9—C8—C7	120.84 (12)	C6—C5—H5	119.8
C9—C8—H8	119.6	C4—C5—H5	119.8
C7—C8—H8	119.6	C2—C3—C4	119.18 (17)
C5—C6—C1	117.74 (14)	C2—C3—H3A	120.4
C5—C6—C7	121.72 (13)	C4—C3—H3A	120.4
C1—C6—C7	120.53 (12)	C14—C19—C18	121.1 (2)
C8—C9—C10	118.94 (13)	C14—C19—H19	119.5
C8—C9—C13	126.17 (13)	C18—C19—H19	119.5
C10—C9—C13	114.88 (12)	C14—C15—C16	120.9 (2)
O2—C12—O3	124.45 (14)	C14—C15—H15	119.6
O3—C12—C11	123.16 (12)	C16—C15—H15	119.6
O2—C12—C11	112.38 (13)	C16—C17—C18	118.69 (19)
N2—C11—C12	111.36 (12)	C16—C17—H17	120.7
N2—C11—H11A	109.4	C18—C17—H17	120.7
C12—C11—H11A	109.4	C3—C4—C5	121.14 (16)
N2—C11—H11B	109.4	C3—C4—H4	119.4
C12—C11—H11B	109.4	C5—C4—H4	119.4
H11A—C11—H11B	108.0	C17—C18—C19	120.5 (2)
C15—C14—C19	117.36 (16)	C17—C18—H18	119.8
C15—C14—C13	120.40 (17)	C19—C18—H18	119.8
C19—C14—C13	122.19 (17)	C17—C16—C15	121.5 (2)
C2—C1—C6	121.30 (14)	C17—C16—H16	119.3
C2—C1—H1	119.4	C15—C16—H16	119.3

### (II) Hydrogen-bond geometry (Å, º)

**Table:**

*D*—H···*A*	*D*—H	H···*A*	*D*···*A*	*D*—H···*A*
C11—H11*B*···O1	0.97	2.39	2.7325 (19)	100
O2—H3···O3^i^	0.82	1.84	2.6599 (16)	177
C5—H5···O3^ii^	0.93	2.40	3.280 (2)	159
C11—H11*A*···O1^iii^	0.97	2.47	3.2814 (19)	141

Symmetry codes: (i) −*x*+2, −*y*+1, −*z*+1; (ii) −*x*+1, −*y*+1, −*z*+1; (iii) *x*, −*y*+1/2, *z*−1/2.
